# Opioidergic modulation of stress-induced hyperalgesia in adult zebrafish

**DOI:** 10.1007/s00702-026-03159-3

**Published:** 2026-05-26

**Authors:** Fabiano V. Costa, Lana Ferreira, Lucca K. Lima, Julia Canzian, Allan V. Kalueff, Denis B. Rosemberg, Carla D. Bonan

**Affiliations:** 1 Graduate Program in Cellular and Molecular Biology, School of Health and Life Sciences, Pontifical Catholic University of Rio Grande Do Sul, Porto Alegre, RS Brazil; 2 Laboratory of Neurochemistry and Psychopharmacology, School of Health and Life Sciences, Pontifical Catholic University of Rio Grande do Sul, Porto Alegre, RS Brazil; 3 Graduate Program in Medicine and Health Sciences, School of Medicine, Pontifical Catholic University of Rio Grande Do Sul, Porto Alegre, RS Brazil; 4 Laboratory of Experimental Neuropsychobiology, Department of Biochemistry and Molecular Biology, Natural and Exact Sciences Center, Federal University of Santa Maria, Santa Maria, RS Brazil; 5 Graduate Program in Biological Sciences: Toxicological Biochemistry, Federal University of Santa Maria, Santa Maria, RS Brazil; 6 Institute of Translational Biomedicine, St. Petersburg State University, St Petersburg, Russia; 7 International Zebrafish Neuroscience Research Consortium (ZNRC), LA New Orleans, USA; 8 Undergraduate Program in Nursing, La Salle University, Canoas, RS Brazil

**Keywords:** Zebrafish, Chronic stress, Nociception, Opioidergic system, Stress-induced sensitization

## Abstract

Depression and pain share overlapping central neurobiological pathways that represent key pharmacological targets in neuropsychiatric and pain research. However, the neuropharmacological mechanisms by which unpredictable chronic stress (UCS) modulates nociception in translational vertebrate models remains poorly understood. Here, we characterized a zebrafish model of stress-induced sensitization to a visceral chemical challenge by exposing adult fish to a 7- or 14-day UCS protocol (UCS7/UCS14) followed by intraperitoneal acetic acid (AA) injection (1.0–5.0% v/v). In unstressed fish, AA at 1.0% remained subthreshold for inducing the characteristic writhing-like body curvature endpoint, whereas at 5.0% produced a robust response, hence validating the assay. In contrast, UCS exposure revealed a marked nociception-like response to AA 1.0%, reflected by increased body curvature index, indicating a lowered response threshold after chronic stress. Locomotor endpoints exhibited stress-dependent cross-over effects: AA at 1.0% decreased distance traveled in unstressed fish but increased locomotion and reduced immobility in UCS-exposed fish. Pharmacological validation (in UCS7) showed that morphine attenuated the UCS-sensitized body curvature response, whereas diclofenac did not, consistent with an opioidergic contribution to the nociception-like endpoint. Whole-body cortisol levels were elevated by UCS exposure (with or without AA 1.0%) and reduced by morphine in the UCS + AA condition, whereas diclofenac was inactive. Together, these findings establish a pharmacologically tractable adult zebrafish model of stress-induced sensitization with translational relevance for CNS-targeted analgesic discovery and mechanistic studies of stress–pain comorbidity.

**Supplementary Information:**

The online version contains supplementary material available at 10.1007/s00702-026-03159-3.

## Introduction

Affective disorders and pain have long been recognized as clinically comorbid conditions (Han and Pae 2015), with chronic pain worsening depressive symptoms, and depression, in turn, intensifying pain perception (Hooten 2016; Yao et al. 2023). Clinical studies further suggest that individuals experiencing chronic stress are more susceptible to developing chronic pain disorders, including fibromyalgia and irritable bowel syndrome, highlighting the critical role of stress and stress-related central nervous system (CNS) deficits in pain perception (Borsook et al. 2012; Vachon-Presseau et al. 2013). Importantly, shared pathways of stress and pain involve central monoaminergic and opioidergic neurotransmission systems that represent key pharmacological targets in neuropsychiatric and analgesic therapies and play a crucial role in modulating mood and pain (Haase and Brown 2015; Sheng et al. 2017). The overlap of these neural circuits, especially the limbic system responsible for both emotional- and pain-processing responses, further supports the integrated nature of their pathogenesis (Meerwijk et al. 2013; Sheng et al. 2017). Chronic neuroinflammation, a common feature of both depression and certain types of pain, also contributes to these overlapping pathogenetic mechanisms (Dooley et al. 2018; Lee and Giuliani 2019; Walker et al. 2014).

Animal experimental models are essential tools for investigating the neuropathological mechanisms underlying stress–pain interactions (Ulrich-Lai and Herman 2009). A common protocol used to assess depression-like states in animal models involves the exposure to chronic stress, such as the unpredictable chronic stress (UCS) (Burstein and Doron 2018; Piato et al. 2011). Based on repeated exposure to varied and unpredictable stressors, the rodent UCS model has been widely used to mimic depression-related behaviors and neurochemical changes observed in humans (Willner 2017). These neurobehavioral effects of UCS are typically associated with the dysregulation of the hypothalamic-pituitary-adrenal (HPA) axis, increased pro-inflammatory cytokines, and impaired serotonergic function, all of which contribute to both depressive symptoms and heightened pain sensitivity (hyperalgesia) (Burke et al. 2017; Slavich and Irwin 2014).

Complementing rodent models, a small freshwater teleost fish, the zebrafish (*Danio rerio*), provides a scalable vertebrate platform for neuropharmacological investigations of complex CNS disorders, including depression and pain (Kysil et al. 2017; Stewart et al. 2014). The UCS paradigms have successfully been adapted for zebrafish, demonstrating overt behavioral, neurochemical and genomic phenotypes that generally resemble those observed in rodent stress models (Piato et al. 2011; Wong et al. 2010). Indeed, UCS-exposed zebrafish exhibit anxiety-like behaviors, reduced exploration and altered monoaminergic neurotransmission, paralleling rodent and clinical findings (Kysil et al. 2017; Maximino et al. 2010). Since stress-induced changes affect mood-related behaviors (Oliveira et al. 2013; Piato et al. 2011), whereas mood and pain share common pathogenetic pathways (De Ridder et al. 2021; Han and Pae 2015; Yang and Chang 2019), stress may impact pain sensitivity, hence supporting the interplay between chronic stress, depression, and pain. Despite the growing interest in zebrafish models for studying pain and depression (Costa et al. 2019b, 2022; Kalueff et al. 2014), the relationship between stress and pain responses in these fish remains poorly understood. However, it may help clarify the evolutionary conserved link between pain- and depression-like phenotypes across vertebrates. Thus, we hypothesize that unpredictable chronic stress (UCS) may produce a pharmacologically tractable hyperalgesic phenotype mediated by central mechanisms in zebrafish.

Understanding these mechanisms is critical for developing CNS-targeted pharmacological strategies that address the stress–pain comorbidity. Here, we pharmacologically characterize a zebrafish model of stress-induced hyperalgesia using centrally and peripherally acting analgesics to dissociate underlying mechanisms. The present study applied a 7–14-day UCS paradigm to induce depression-like phenotypes in zebrafish, followed by intraperitoneal administration (i.p.) of a nociceptive agent (1–5% acetic acid, AA) and quantifying pain-like behaviors using the aquatic writhing assay. To explore the underlying mechanisms of UCS-induced hyperalgesia, we further examined the effects of morphine, a centrally acting analgesic, and diclofenac, a peripheral anti-inflammatory agent. This combined protocol allows for the concurrent assessment of depression- and pain-related responses in the same organism, providing a translationally relevant platform to investigate shared mechanisms underlying affective and nociceptive processes and to distinguish central versus peripheral modulation of stress-induced hyperalgesia.

## Methods

### Animals

A total of 300 adult wild-type zebrafish (AB strain; 5–7 months old; ~50:50 male: female) were maintained in automated recirculating systems (ZebTEC, Tecniplast, Italy) with reverse-osmosis-filtered water and conditions, as in (Westerfield 1993), for at least 2 weeks prior to the beginning of experiments. Since no sex differences were reported for AA-induced nociception-like endpoints in adult zebrafish (Costa et al. 2019b) or for whole-body cortisol responses in our previous work (Costa et al. 2023), data from males and females were pooled to maximize statistical power and reduce animal use, in accordance with the 3Rs principles. Animals were obtained from an in-house breeding colony and maintained under standard conditions at the authors’ facility. Fish were maintained at 28℃ ± 2℃, pH 7.0–7.5, water conductivity 300–700 µS, ammonia < 0.02 mg/L, hardness 80–300 mg/L, nitrite < 1 mg/L, nitrate < 50 mg/L, and chloride 0 mg/L, under a 14 h light:10 h dark photoperiod cycle (lights on: 07:00 am) and fed with commercial flakes (TetraMin Tropical Flake Fish™) three times a day. Water pH and conductivity were monitored daily, and nitrogen compounds were measured weekly, in accordance with established zebrafish husbandry standards (Alestrom et al. 2020). All protocols were approved by the Ethics Committee on the Use of Animals (CEUA, protocol number 11607) and animal experimentation fully adhered to the National Institutes of Health Guide for the Care and Use of Laboratory Animals and the guidelines of the National Council for the Control of Animal Experimentation (CONCEA). The AB fish were selected here as a common zebrafish strain widely used in neurobehavioral assays (Bertoncello et al. 2024).

### Chemicals

The following chemicals were used in the present study: acetic acid (AA; Merck KGaA, Darmstadt, Germany, CAS No. 64-19-7), morphine sulfate (MOR; Sigma-Aldrich, St. Louis, USA, CAS No. 6211-15-0), phosphate buffered saline (PBS; Sigma-Aldrich), and diclofenac sodium (DS; Novartis, São Paulo, Brazil; CAS No. 15307-79-6).

### Unpredictable chronic stress protocol (UCS)

Following a two-week acclimation period, the UCS was performed based on a protocol described previously, with some modifications (Piato et al. 2011). Briefly, fish were submitted twice a day to various stressors for 7 or 14 consecutive days (UCS7 or UCS14), as summarized in Table 1. The UCS stressor battery included: *Change of environments*: keeping the tanks surrounded by a different color from the animal’s natural environment (green, yellow, red) for 6 h; *Heating*: increasing the water temperature of the tank up to 33 °C for 30 min; *Social isolation*: animals were placed individually in a 250 mL beaker for 6 h; *Cooling*: cooling the tank water down to 23 °C for 30 min; *Crowding*: placing a group of 10 animals for 50 min in a 250 mL beaker; *Low water level*: decreasing the water level in housing tanks until the animals’ dorsal body walls are exposed for 2 min; *Changing water*: animals were kept in the tank while the water was changed three consecutive times; *Changing tanks*: animals were transferred to a fresh tank three times; *Net chasing*: chasing animals for 8 min with a net. Aeration and temperature were controlled during each stressor presentation (except during heating and cooling stress). To prevent habituation and maintain stress unpredictability, the time and sequence of stressor presentations were changed daily. An unstressed control group remained intact in the same room for 7 or 14 days, respectively.

**Table 1:** Unpredictable chronic stress protocol

Week	Thursday	Friday	Saturday	Sunday	Monday	Tuesday	Wednesday
Week 1	Water change	Social isolation	Crowding	Low water level	Heating	Net chasing	Crowding
	Heating	Cooling	Change environment	Tank change	Cooling	Change environments	Social isolation
Week 2	Low water level	Tank change	Heating	Social isolation	Net chasing	Cooling	Heating
	Cooling	Crowding	Water change	Change environments	Low water level	Tank change	Crowding
Week 3	Behavioral analysis						

### Experimental design, group allocation, and intraperitoneal (i.p.) injections

**Fig. 1:**
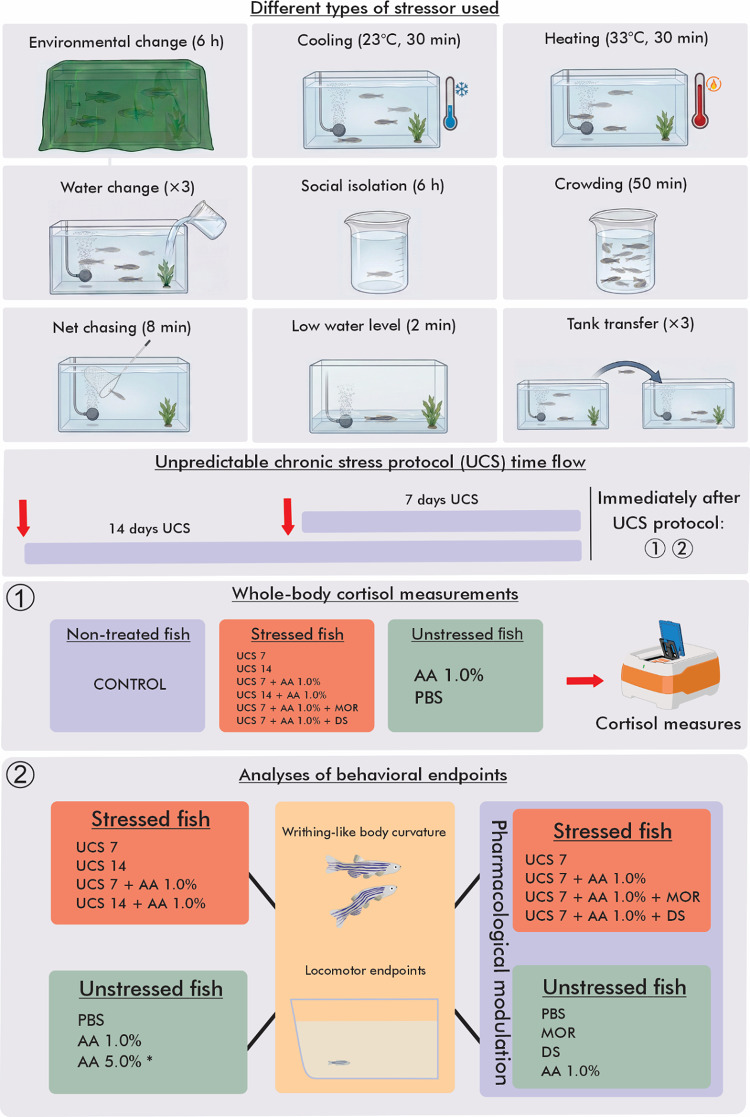
Schematic overview of the unpredictable chronic stress (UCS) protocol and experimental workflow in adult zebrafish. Animals were acclimated and then exposed to UCS for 7 days (UCS7) or 14 days (UCS14), consisting of two daily stressors presented in a randomized order (see Table 1). Immediately after UCS protocol, fish received intraperitoneal injections of PBS (vehicle) or acetic acid (AA; 1.0%), with AA 5.0% used only in unstressed fish for assay validation due to tolerability constraints under UCS. Whole-body cortisol was quantified in an independent cohort subjected to the same experimental design and sampled immediately after treatments for extraction and ELISA. For pharmacological validation (UCS7), diclofenac (DS; 40 mg/kg, i.p.) was administered 15 min prior to AA 1.0%, and morphine (MOR; 2.5 mg/kg, i.p.) was co-administered immediately before AA 1.0%. Behavioral endpoints (distance traveled, immobility, and writhing-like body curvature) were recorded for 6 min after fish regained postural equilibrium. * The animals injected with AA 5.0% were used only in the body curvature index analyses. Created with BioRender

Zebrafish (*n* = 8–10 per group) were randomly selected from at least three housing tanks (for non-stressed controls) or from the respective UCS batches (UCS7 or UCS14). Sample size was selected based on our previous zebrafish AA studies (Costa et al. 2019a, b) and consistent with similar experimental designs in adult zebrafish nociception/stress research, while minimizing animal use. A schematic timeline (chronogram) summarizing UCS exposure, injection timing, behavioral recording, and cortisol measure is provided in Fig. 1.

#### Acute AA challenge after UCS

Immediately after UCS7 or UCS14 (see Sect. "Unpredictable chronic stress protocol (UCS)"), fish received a single i.p. injection of either PBS (vehicle) or AA (1.0% vol/vol in PBS). In this context, UCS refers to the chronic stress protocol (7 or 14 days) previously applied to the animals. Additionally, a group of unstressed animals was injected with AA 1.0% or 5.0% alone, described as positive control. The AA concentrations were selected based on dose-dependent nociception-like responses in adult zebrafish, where AA 5.0% reliably elicits writhing-like body curvature responses, while AA 1.0% is frequently subthreshold in unstressed fish (Costa et al. 2019b; Sneddon et al. 2003; Taylor et al. 2017).

#### Injection procedure

Fish were gently handled and briefly anesthetized in cold water (< 5 s), as previously described (Kinkel et al. 2010). The animals were immobilized using a small wet net and injected intraperitoneally through the midline between the pelvic fins. All injections used a BD Ultra-Fine™ 30U syringe (6 × 0.25 mm needle) with 10 µL, a volume previously shown not to impair zebrafish behavior (Kinkel et al. 2010; Richetti et al. 2011). The injection was completed within ~ 5 s. Cold-water anesthesia and the brief handling/injection procedure were used to avoid potential confounding effects of chemical anesthetics (e.g., tricaine) on locomotor and nociception-like endpoints during acute post-injection recordings. Importantly, this protocol allows a fast evaluation of the swimming activity after fish return to the water, minimizing potential pharmacological interference on complex behaviors (e.g., immobility and locomotion). After the injection, the fish were returned immediately to their beakers. Behavioral recording began once fish regained postural equilibrium (~ 1 min post-injection), enabling assessment of acute AA-induced behavioral responses (Costa et al. 2019b). Sex was not used as an analytical factor here, and males/females were pooled, as described in Sect. "Animals".

### Pharmacological modulation of the UCS + AA phenotype

Zebrafish (*n* = 8–10 per group) for this experiment were randomly selected from at least three housing tanks (non-stressed controls) or from UCS7 batches. The UCS7 model was used for pharmacological experiments because UCS7 and UCS14 produced similar results in the initial protocol, allowing for the refinement and reduction of animal use. Animals were allocated to the following groups: (i) Controls (no UCS): PBS, MOR, DS, AA 1.0%; (ii) UCS exposure: UCS7 + PBS; (iii) Stress-sensitized condition: UCS7 + AA 1.0%; (iv) Pharmacological modulation: UCS7 + AA 1.0% + MOR; UCS7 + AA 1.0% + DS. Group presentation in figures and statistical analyses followed a factorial structure (Stress exposure × Condition), as detailed in Sect. "Statistical analyses and data handling".

#### Drug administration

Injection procedures followed Sect. "Injection procedure". Diclofenac pretreatment: UCS7 fish received DS (40 mg/kg, i.p.) 15 min prior to AA 1.0% injection. Morphine co-administration: UCS7 fish received MOR (2.5 mg/kg, i.p.) immediately before AA 1.0% injection. Diclofenac was selected as a clinically approved nonsteroidal anti-inflammatory drug (Altman et al. 2015), widely used to describe inflammatory contributions to nociception-related phenotypes. Morphine was selected as a clinically approved opioid analgesic (Pathan and Williams 2012), widely used in experimental pain models (Bjorkman et al. 1992; Hamann et al. 2016; Llorca-Torralba et al. 2018; Rodrigues-Filho et al. 2004) and known to modulate nociception-like behavior in zebrafish (Costa et al. 2019b; Taylor et al. 2017). Drug doses were selected based on established zebrafish protocols (Costa et al. 2019b; Sneddon et al. 2003; Taylor et al. 2017).

### Behavioral recording and endpoints

#### Recording conditions and timing

Behavioral testing was performed between 12:00 and 16:00. Fish were fasted on the day of the experiments (food withheld before testing) to reduce variability from feeding-related arousal and gastrointestinal distension, which can influence locomotor activity and abdominal posture during short recordings. Water conditions during behavioral testing matched those of the housing tanks. Immediately following the i.p. injection(s), fish were individually transferred to observation tanks (15 × 13 × 10 cm; length × height × width) with a 10-cm water depth and recorded for 6 min using a digital camera (webcam Ultra HD Logitech^®^ 4 K PRO, Lausanne, Switzerland). Behavioral recordings began once the fish regained equilibrium (~ 1 min post-injection).

#### Locomotor endpoints

All behavioral analyses were performed offline by experimenters blinded to group allocation, using the EthoVision^®^ XT11.5 software (Noldus IT, Wageningen, Netherlands) at 30 frames/s. Distance traveled (cm) and immobility duration (s) were quantified based on the center body point coordinates. Immobility was defined as complete immobility (< 0.59 cm/s) except small movements of fins and eyes, accompanied by fast opercular beat rates, as described elsewhere (Wiprich et al. 2020). Distance traveled and immobility were treated as locomotor/freezing-like endpoints, whereas body curvature was treated as a nociception-like endpoint (see below). All experiments were performed as planned, and all analyses and all endpoints assessed were included without omission.

#### Writhing-like body curvature

Writhing-like behavior represents abnormal body constriction/curvature following AA injection and was defined according to the Zebrafish Behavioral Catalogue (ZBC) (Kalueff et al. 2013, 2025). A key part of zebrafish pain-related behaviors (ZBC1 term 1.104), writhing-like behavior (ZBC2 term 2.121) represents an abnormal constriction of body that is often seen after the administration of AA, reflecting acute discomfort, stress and nociception-like state (Kalueff et al. 2025). Briefly, to assess this phenotype, screenshots of the animal’s sagittal plane were taken every 30 s (6 min = 12 screenshots), and later analyzed using ImageJ 1.45 for Windows (National Institutes of Health, NIH, Bethesda, USA). Three points were selected (frontal, central, posterior) to estimate curvature angle. Angles were subtracted from 180° and multiplied by (−1) to estimate the body curvature index. The area under the curve (AUC) was calculated to quantify the total body curvature index over time. Inter-rater reliability was assessed by two trained observers blinded to condition (inter-rater reliability > 0.90). A representative image illustrating differences in body curvature is shown in Supplementary Fig. 1, and a representative heatmap of locomotor profiles is shown in Supplementary Fig. 2S. After the 6-min behavioral recording, animals were anesthetized in cold water (4 °C) and euthanized by decapitation.

### Whole-body cortisol measurements

Whole-body cortisol was assessed in an independent cohort of zebrafish subjected to the same experimental design (UCS exposure and AA/drug treatment) as described above, but not used for behavioral recording. For each experimental condition, an independent cohort (*n* = 8–10 per group) was used for cortisol determination. For cortisol analysis, fish were euthanized immediately after the injection protocol (matching the timing of the behavioral experiment) and rapidly frozen in liquid nitrogen for 20–30 s prior to extraction. Whole-body cortisol was extracted following the ether-based extraction protocol (Mezzomo et al. 2019) and quantified in duplicates using a commercially enzyme-linked immunosorbent assay kit (EIAgen™ Cortisol test, BioChem ImmunoSystems) (Sink et al. 2008). Results were expressed as ng cortisol/g tissue. A strong positive correlation was observed (R² = 0.9413), and inter- and intra-assay coefficients of variation values were low (7–10% and 5–9%, respectively).

### Statistical analyses and data handling

Data normality and homogeneity of variances were assessed by the Kolmogorov–Smirnov and Bartlett’s tests, respectively. Primary analyses were performed using factorial models aligned with the experimental design. Specifically, endpoints were analyzed using two-way ANOVA with factors Stress exposure (Control vs. UCS) and Condition (e.g., PBS vs. AA 1.0% in factorial comparisons), followed by appropriate multiple-comparison procedures (Bonferroni correction) for pre-specified simple-effects contrasts (e.g., within-stress comparisons and between-stress comparisons under the same condition). Dose-validation analyses in unstressed fish (e.g., PBS vs. AA 1.0% vs. AA 5.0%) were also reported explicitly where the UCS-matched dose level was not available for factorial comparison. Pharmacological validation and cortisol data were analyzed by one-way analysis of variance (one-way ANOVA; factor: group), followed by Tukey’s multiple-comparison test when appropriate. Results were expressed as means ± standard error of the mean (S.E.M.). Immobility duration was log-transformed to meet assumptions of normality, and the analyses were then performed on transformed data. The inter-rater reliability was assessed by Spearman’s rank correlation (*r* > 0.85). Statistical analysis was performed using the GraphPad Prism10 software (GraphPad Software, Boston, USA). All fish tested were included in the final analysis without attrition or exclusion, and all planned analyses are presented. All behavioral quantification and statistical analyses were performed by experimenters blinded to group allocation.

## Results

### UCS exposure induces a nociception-like response to AA 1.0%

**Fig. 2:**
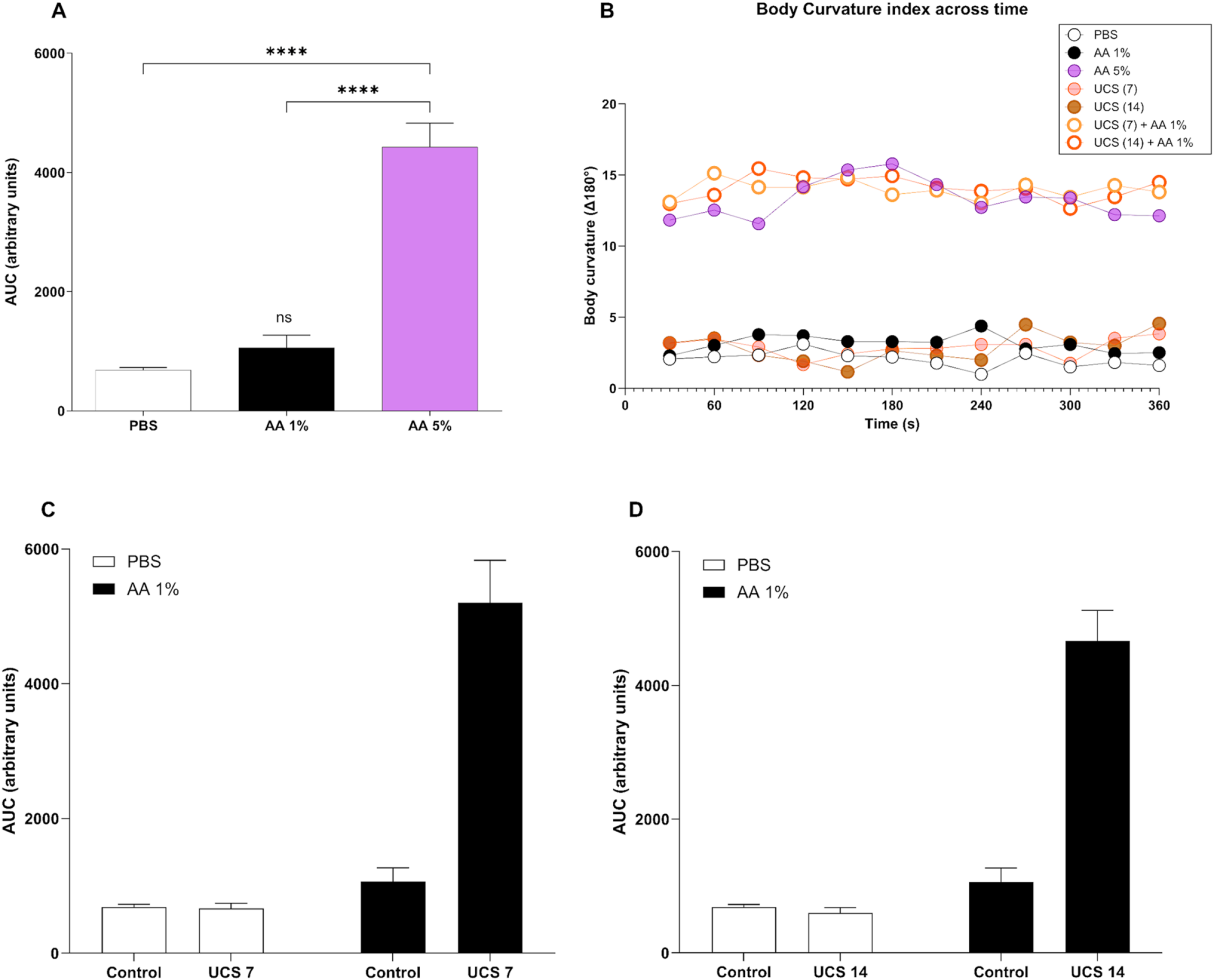
Stress exposure reveals a nociception-like response to acetic acid (AA) 1.0% in adult zebrafish. **A **Concentration validation in unstressed fish: area under the curve (AUC) of the body curvature index following phosphate-buffered saline (PBS vehicle), AA 1.0%, or AA 5.0% (one-way ANOVA followed by Tukey’s test; (****p < 0.0001 vs. AA 5.0%). **B** Body curvature index across time (30-s) in Control and UCS-exposed fish injected with PBS or AA 1.0% and AA 5.0% (shown for visualization of dynamics). **C **UCS7: AUC analyzed by two-way ANOVA (Stress: Control vs. UCS7; Condition: PBS vs. AA 1.0%) followed by the Bonferroni post-hoc test. **D** UCS14: AUC analyzed by two-way ANOVA (Stress: Control vs. UCS14; Condition: PBS vs. AA 1.0%) followed by the Bonferroni post-hoc test. Data are expressed as means ± S.E.M.; *n* = 8–10 per group. UCS refers to the chronic stress protocol (7 or 14 days)

In unstressed zebrafish, AA 1.0% did not significantly increase the AUC of the body curvature index compared with PBS controls, whereas AA 5.0% induced a robust increase in AUC, confirming assay responsiveness in controls (Fig. 2A, B). Because AA 5.0% was not evaluated in the UCS-exposed fish, factorial analyses were restricted to AA 1.0% (PBS vs. AA 1.0%) across the stress exposure groups.

For the nociception-like endpoint (body curvature AUC), the two-way ANOVA revealed significant Stress × Condition interactions following both UCS7 (F_(1,29)_ = 50.40, *p* < 0.0001) and UCS14 (F_(1,29)_ = 64.44, *p* < 0.0001) (Fig. 2C, D). In unstressed fish, AA 1.0% did not significantly change AUC compared with PBS controls (UCS7, *p* = 0.6590; UCS14, *p* = 0.4315). Likewise, no differences between Control and UCS groups were observed under PBS conditions (UCS7, *p* > 0.9999; UCS14, *p* > 0.9999). In contrast, AA 1.0% produced a marked increase in AUC in zebrafish previously exposed to UCS (UCS7: UCS + AA vs. UCS + PBS, *p* < 0.0001; UCS14: UCS + AA vs. UCS + PBS, *p* < 0.0001), and under AA 1.0% conditions UCS-exposed fish exhibited significantly higher AUC than unstressed fish (UCS7: Control + AA vs. UCS + AA, *p* < 0.0001; UCS14: Control + AA vs. UCS + AA, *p* < 0.0001), indicating stress-induced sensitization to a subthreshold nociceptive challenge.

**Fig. 3:**
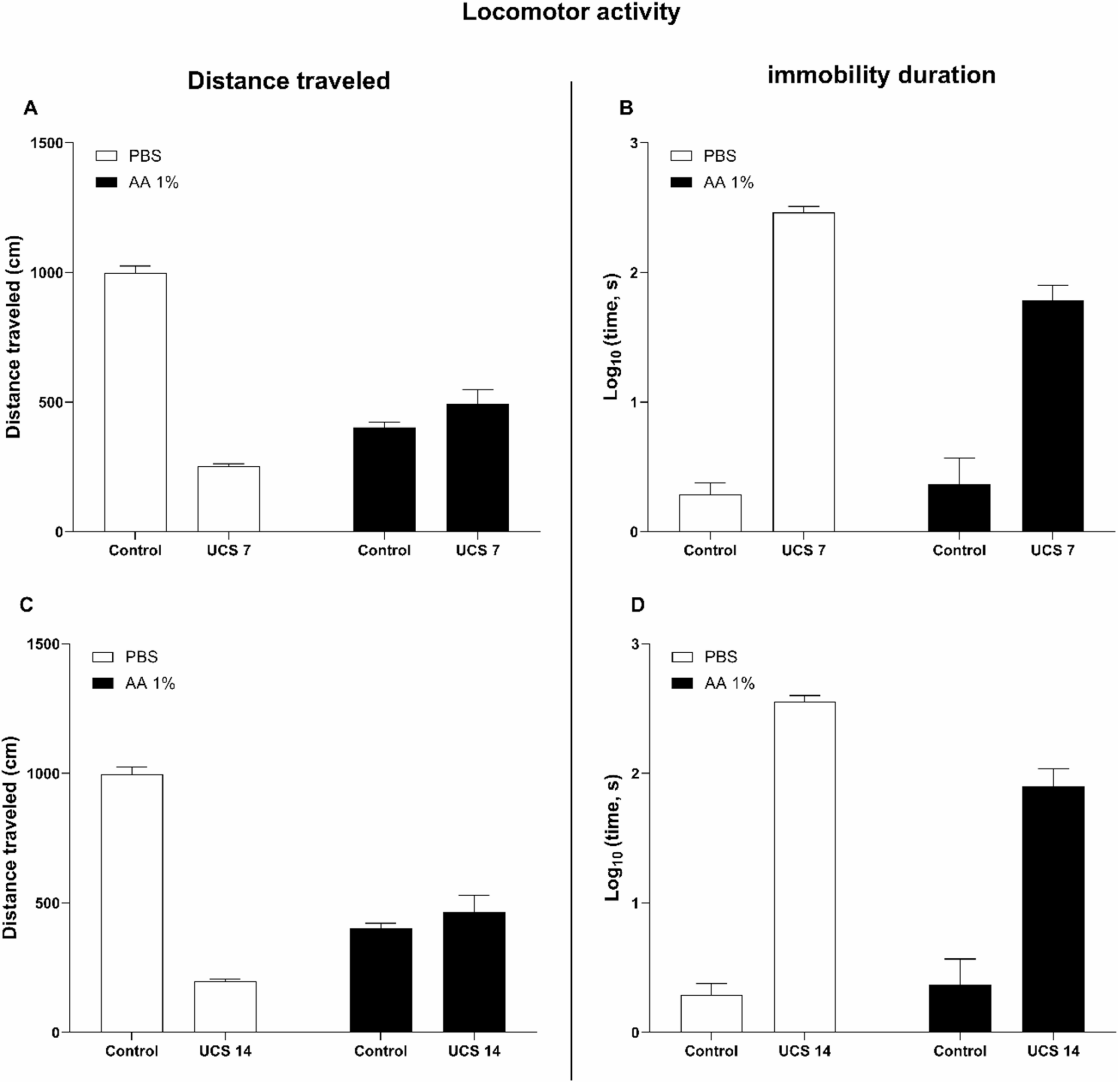
Locomotor activity following UCS exposure and acetic acid (AA) 1.0% challenge in adult zebrafish. **A** Distance traveled (cm) and **B** immobility duration (log_10_-transformed, s) in Control and UCS7-exposed fish injected with phosphate-buffered saline (PBS) or AA 1.0%. **C** Distance traveled and **D** immobility duration in the Control and UCS14-exposed fish injected with PBS or AA 1.0%. Data were analyzed by the two-way ANOVA (Stress: Control vs. UCS; Condition: PBS vs. AA 1.0%) followed by the Bonferroni post-hoc test. Data are expressed as means ± S.E.M.; *n* = 8–10 per group. UCS refers to the chronic stress protocol (14 days)

Locomotor activity also displayed robust Stress × Condition interactions. For distance traveled (Fig. 3A–C), the two-way ANOVA showed strong interactions following both UCS7 (F_(1,36)_ = 157.5, *p* < 0.0001) and UCS14 (F_(1,36)_ = 136.0, *p* < 0.0001) protocols. After PBS injection, UCS markedly reduced distance traveled compared with controls (UCS7, *p* < 0.0001; UCS14, *p* < 0.0001). In unstressed fish, AA 1.0% significantly reduced distance traveled relative to PBS (UCS7, *p* < 0.0001; UCS14, *p* < 0.0001). In contrast, UCS-exposed fish plus AA 1.0% significantly increased distance traveled compared with UCS + PBS (UCS7, *p* < 0.0001; UCS14, *p* < 0.0001), eliminating the difference between Control and UCS under AA 1.0% conditions (UCS7, *p* = 0.1309; UCS14, *p* = 0.4884). Thus, AA 1.0% produced opposite directional effects on locomotion depending on previous stress exposure (cross-over interaction).

Immobility duration similarly showed significant Stress × Condition interactions following UCS7 (F_(1,36)_ = 9.187, *p* = 0.0045) and UCS14 (F_(1,36)_ = 7.975, *p* = 0.0077) (Fig. 3B–D). The UCS exposure significantly increased immobility relative to controls under both PBS and AA 1.0% conditions (*p* < 0.0001). In unstressed fish, AA at 1.0% did not significantly change immobility (UCS7, *p* > 0.9999; UCS14, *p* > 0.9999). However, UCS-exposed fish plus AA 1.0% significantly reduced immobility vs. UCS + PBS (UCS7, *p* = 0.0010; UCS14, *p* = 0.0021), consistent with a partial shift from stress-induced freezing-like behavior toward increased locomotor activity during nociceptive challenge. Because both UCS7 and UCS14 produced similar patterns across nociception-like (AUC) and locomotor endpoints (distance and immobility), UCS7 was selected for subsequent pharmacological experiments to reduce animal use.

### Morphine, but not diclofenac, attenuates the UCS-sensitized nociception-like response to AA 1.0%

**Fig. 4:**
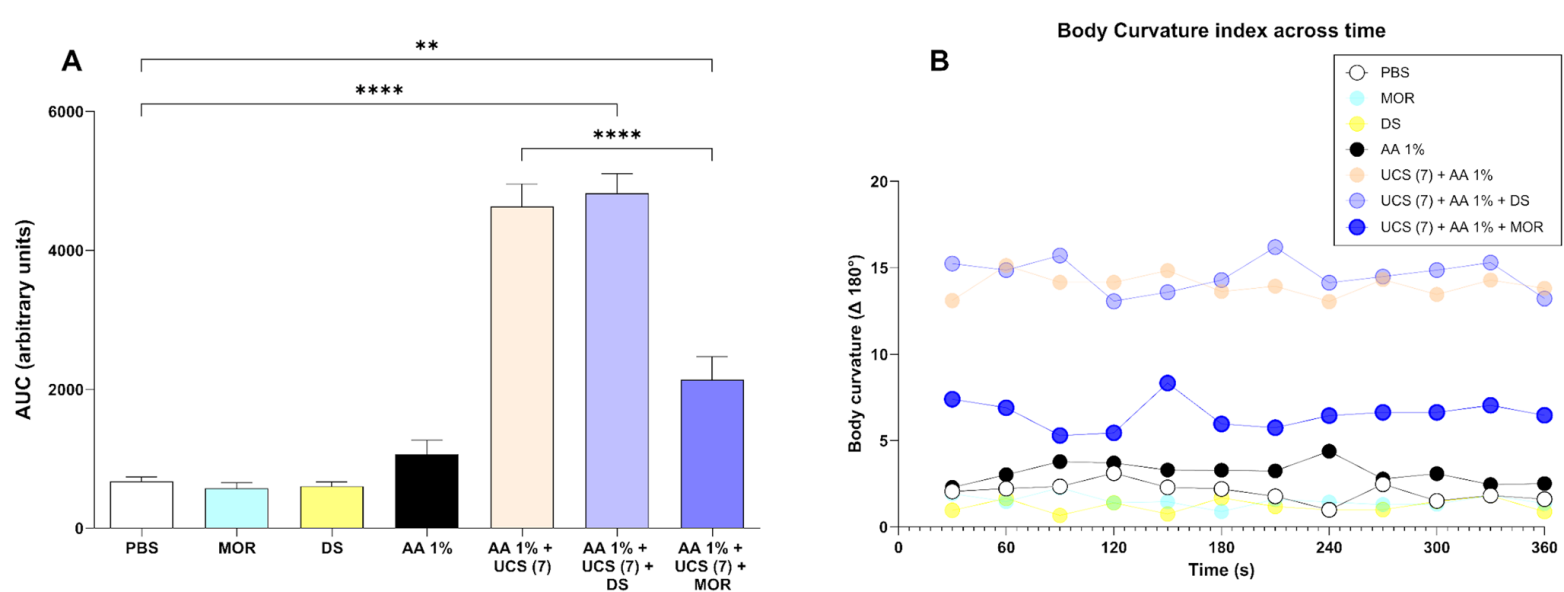
Pharmacological modulation of UCS-induced sensitization to AA 1.0% in adult zebrafish. **A** AUC of the body curvature index in baseline control groups (PBS, morphine/MOR, diclofenac sodium/DS), unstressed fish injected with AA 1.0%, and UCS7-exposed fish challenged with AA 1.0% with or without diclofenac pretreatment (40 mg/kg, i.p.) or morphine co-administration (2.5 mg/kg, i.p.). Data were analyzed by the one-way ANOVA followed by the Tukey’s multiple-comparison test (**p < 0.005; ****p < 0.001). **B** Body curvature index across the 6-min recording period (30-s) for the same experimental groups (shown to visualize temporal response profiles). Data are expressed as means ± S.E.M.; *n* = 8–10 per group. UCS refers to the chronic stress protocol (7 days)

Zebrafish exposed to UCS7 + 1.0% AA showed significantly higher AUC of the body curvature index compared to baseline control groups (PBS, MOR, and diclofenac) and unstressed animals injected with AA 1.0% (Fig. 4A). One-way ANOVA revealed significant differences among groups (F_(6,39)_ = 52.48; *p* < 0.0001). While diclofenac pretreatment did not prevent the UCS7 + plus AA-induced AUC increase, morphine co-administration significantly reduced AUC compared to both UCS7 + AA 1.0% and UCS7 + AA 1.0% + diclofenac groups (Fig. 4A). The time course of the body curvature index across the 6-min recording period is shown in Fig. 4B to visualize response dynamics, with morphine displaying an attenuated profile relative to UCS7 + AA 1.0%, whereas diclofenac did not alter the temporal pattern.

**Fig. 5:**
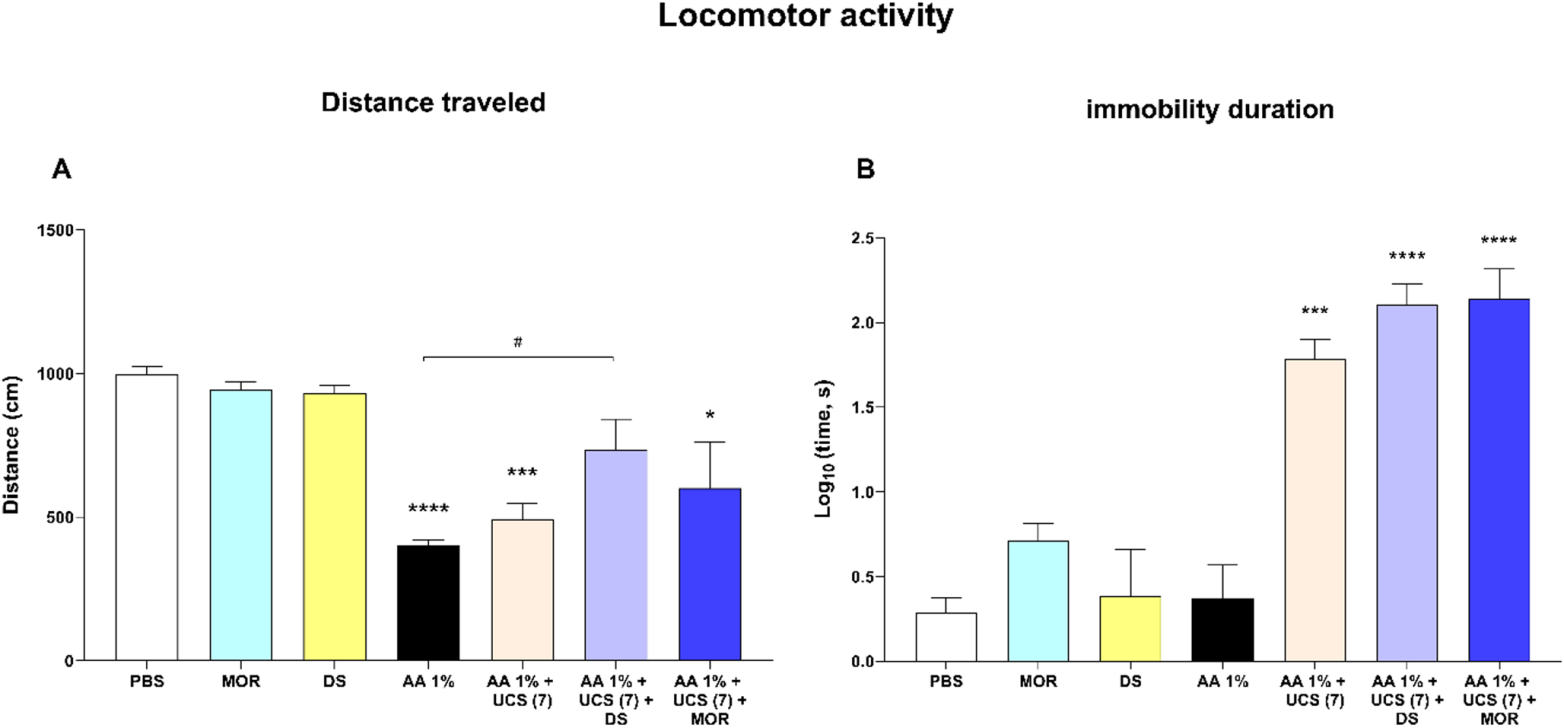
Effects of morphine (MOR, 2.5 mg/kg, i.p.) and diclofenac sodium (DS, 40 mg/kg, i.p.) on chronic stressed animals (UCS; 7 days) following 1.0% acetic acid (AA)-induced changes in zebrafish locomotor behavior. Locomotor endpoints were assessed by distance traveled (**A**) and immobility duration (**B**). Data are expressed as means ± S.E.M. and analyzed by the one-way ANOVA (factor: treatment), followed by the Tukey’s post-hoc test for significant ANOVA data (*p < 0.05 vs. PBS; ***p < 0.005 vs. PBS; ****p < 0.001 vs. PBS; ^**#**^*p* < 0.05 vs. AA 1.0%; *n* = 8–10 per group)

Although both morphine and diclofenac partially rescued the distance traveled in UCS7 + 1.0% AA-exposed zebrafish (Fig. 5A; F_(6,46)_ = 6.35; *p* < 0.0001), they did not reverse the increased immobility duration in UCS7 + 1.0% AA-exposed zebrafish (Fig. 5B). A representative heatmap of adult zebrafish locomotor profiles is shown in Supplementary Fig. 2S.

### Morphine, but not diclofenac, reduces the whole-body cortisol levels in zebrafish exposed to UCS following acetic acid

**Fig. 6:**
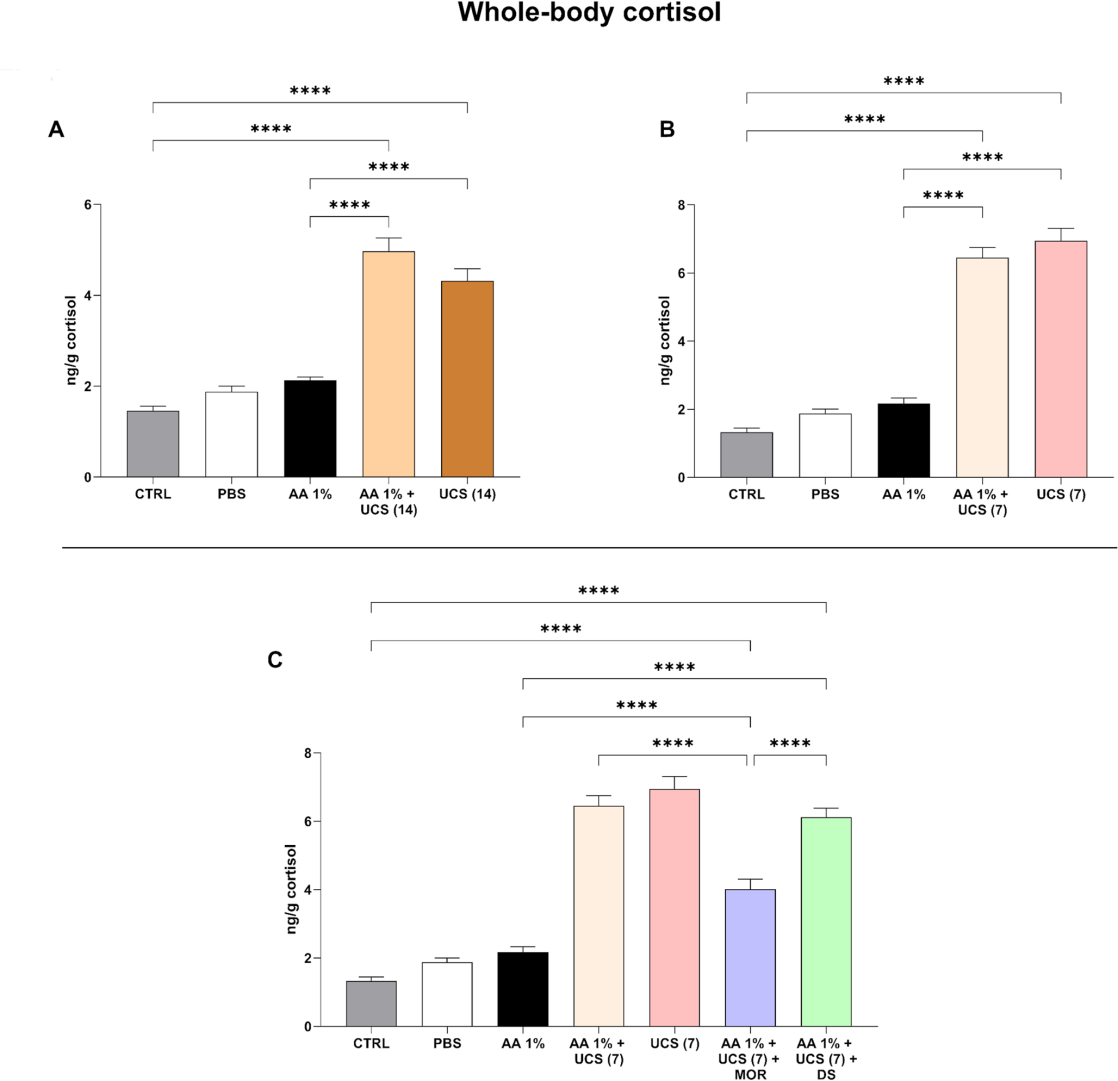
Whole-body cortisol levels in adult zebrafish following unpredictable chronic stress (UCS; 7 days) exposure and pharmacological modulation. **A** Whole-body cortisol (ng/g tissue) in unstressed control groups (CTRL, phosphate-buffered saline/PBS, acetic acid/AA 1.0%) and in UCS14-exposed fish with or without AA 1.0% challenge (AA 1.0% + UCS14; UCS14). **B** Whole-body cortisol (ng/g tissue) in unstressed control groups (CTRL, PBS, AA 1.0%) and in UCS7-exposed fish with or without AA 1.0% challenge (AA 1.0% + UCS7; UCS7). **C** Pharmacological modulation of cortisol in UCS7-exposed animals: effects of morphine co-administration (MOR; 2.5 mg/kg) and diclofenac pretreatment (DS; 40 mg/kg) in the UCS7 + AA 1.0% condition. Cortisol was quantified in independent cohorts subjected to the same experimental procedures. Data were analyzed by the one-way ANOVA followed by the Tukey’s post-hoc multiple-comparison test (****p < 0.001; *n* = 8–10 per group). Data are expressed as means ± S.E.M.; *n* = 7 per group (**A**, **B**) and *n* = 7–9 per group (**C**). Groups included CTRL–utreatednaïve group, *PBS* - control group, *MOR* morphine (2.5 mg/kg), *DS* diclofenac (40 mg/kg), AA − 1% vol/vol

Whole-body cortisol levels significantly differed after both UCS7 or UCS14 exposure (one-way ANOVA: F_(4,30)_ = 134.5, *p* < 0.0001 and F_(4,30)_ = 66.52, *p* < 0.0001, respectively; Fig. 6A, B). Neither PBS injection nor AA 1.0% injection alone change cortisol compared to unstressed controls (UCS7: CTRL vs. PBS, *p* = 0.47; CTRL vs. AA 1.0%, *p* = 0.11; UCS14: CTRL vs. PBS, *p* = 0.55; CTRL vs. AA 1.0%, *p* = 0.12). In contrast, UCS7 or UCS14 exposure significantly increased whole-body cortisol compared with all unstressed control conditions (*p* < 0.0001). Importantly, cortisol levels did not differ between UCS7 and UCS7 plus AA 1.0% groups (*p* = 0.5697) or UCS14 and UCS14 plus AA 1.0% groups (*p* = 0.1534), indicating that stress exposure, rather than AA injection, was the main cause of increased cortisol.

Pharmacological modulation was assessed in UCS7-exposed fish challenged with AA 1.0% (Fig. 6C). The one-way ANOVA revealed significant group differences (F_(6,42)_ = 92.38, *p* < 0.0001). Morphine co-administration significantly reduced whole-body cortisol levels in the UCS7 + AA 1.0% condition compared with UCS7 plus AA 1.0% alone (*p* < 0.0001). In contrast, diclofenac pretreatment did not significantly attenuate cortisol increase in UCS7 +_ AA 1.0% group, remaining similar to the UCS7 and UCS7 + AA 1.0% groups.

## Discussion

In general, the present study demonstrates a pharmacologically sensitive hyperalgesic phenotype induced by chronic stress in adult zebrafish. Notably, UCS exposure induced a robust writhing-like response after AA 1.0% injection, a concentration unaltering the body curvature index in non-stressed fish. In contrast, AA 5.0% produced a clear nociception-like phenotype in controls, consistent with our previous reports (Costa et al. 2019a, b). Interestingly, the response observed in UCS-exposed fish injected with AA 1.0% was similar to unstressed animals injected with AA 5.0%, supporting a stress-induced shift in responsiveness (e.g., lowered response threshold) rather than a nonspecific motor response. Importantly, AA 5.0% was used only in unstressed fish for assay validation, due to tolerability constraints under UCS. Because AA is a chemical irritant used as a nociceptive challenge, we interpret this phenomenon primarily as stress-induced sensitization to a subthreshold nociceptive chemical challenge, and use the term “hyperalgesia/sensitization” to describe the phenotype.

The link between stress and pain has been extensively examined in both rodent and clinical literature. For example, acute stress evokes pronounced stress-induced hypoalgesia (Timmers et al. 2018), likely representing a protective mechanism, allowing individuals to focus on escaping immediate vital threats (Ulrich-Lai and Herman 2009). Unlike acute stress, the effects of chronic stress exposure on pain sensitivity are heterogeneous and context-dependent, varying with stressor type, duration, and outcome measures. For example, chronic stress increases pain in clinical (Timmers et al. 2018) and rodent studies (Liu et al. 2019). Importantly, these effects are strongly modulated by centrally acting pharmacological systems, particularly opioidergic pathways that regulate both stress reactivity and nociceptive processing (Butler and Finn 2009).

Corroborating previous reports (Costa et al. 2019b), AA 1.0% remained subthreshold for the writhing-like curvature endpoint in unstressed fish, whereas UCS exposure here induced a robust response to such injection. We demonstrated that UCS exposure followed by AA 1.0% injection increased the AUC of the body curvature index, and this nociception-like phenotype co-occurred in a stress-dependent manner of locomotor behavior, with AA 1.0% decreasing distance traveled in unstressed fish but increasing locomotion and reducing immobility in UCS-exposed fish. Furthermore, the present study also demonstrated that UCS-induced increases in the writhing-like body curvature endpoint were sensitive to morphine, but not diclofenac.

Since AA 1.0% alone did not alter immobility in unstressed fish, and UCS exposure robustly increased it and reduced distance traveled under PBS conditions, the observed freezing-like phenotype was primarily stress-driven. Notably, AA 1.0% modulated this stress-induced behavioral state by increasing distance traveled and reducing immobility in UCS-exposed fish, indicating a stress-dependent reorganization of locomotor behavior during nociceptive challenge. Lastly, neither morphine nor diclofenac reversed the UCS-associated increase in immobility, despite partially restoring distance traveled. Therefore, these findings support that UCS lowers the response threshold to a nociceptive chemical challenge in adult zebrafish, with differential sensitivity of the primary nociception-like endpoint (curvature AUC) to centrally acting opioidergic modulation.

In zebrafish, genes associated with stress responses show overlapping expression patterns in depression and pain models (de Abreu et al. 2021), which also share common monoaminergic mechanisms (Demin et al. 2020). Chronic stress disrupts several brain regions and neurotransmitters related to pain and mood responses in zebrafish. For instance, the *hypothalamus* plays a crucial role in stress response, regulating dopaminergic and serotonergic pathways (Corradi and Filosa 2021; Martins et al. 2024). The *dorsal raphe* is rich in serotonergic neurons and shows activity modulation during stress (Martins et al. 2024). The *telencephalon*, homologous to the mammalian amygdala, is involved in processing stress and anxiety-related behaviors in fish by altered the glutamatergic neurotransmission (Corradi and Filosa 2021). The *caudal hypothalamus* presents a group of dopaminergic neurons, influencing locomotor and exploratory behaviors, and potentially mediating stress-coping strategies (Corradi and Filosa 2021). Similar to humans, chronic stress-induced dysregulation of these neurotransmitters and brain regions may represent a potential interaction between emotional states and pain perception in zebrafish models. The correction of this sensitized phenotype by morphine, but not diclofenac, supports a predominant role for central neuropharmacological mechanisms, although peripheral pathways cannot be excluded as well. Such contrasting effects of morphine and diclofenac likely arise from distinct mechanisms by which these drugs modulate pain. For instance, in humans, morphine primarily acts through opioid receptors, centrally blocking pain transmission, influencing emotional pain responses, and playing a key role in mood regulation (Reeves et al. 2022). Moreover, the µ-opioid (MOP) and δ-opioid (DOP) receptors, both activated by morphine, produce analgesic effects and impact affective behaviors. For instance, MOP agonists induce euphoria and improve stress coping, while DOP agonists trigger anxiolytic and antidepressant effects (Lutz and Kieffer 2013; Valentino and Volkow 2018).

Like in humans, the zebrafish µ-opioid (zMOP) receptor is widely distributed in brain regions involved in analgesia and mood and produces similar effects (Sivalingam et al. 2020). For instance, the zMOP activation exerts analgesic properties, as morphine prevents pain-like behaviors in both adult and larval zebrafish in inflammatory and visceral pain models, while also inducing addiction (Costa et al. 2019b; Magalhaes et al. 2017; Taylor et al. 2017). Interestingly, similar to humans, adverse effects can also be observed following zMOP activation with different agonists, including sedation (mitragynine and morphine) (Cachat et al. 2010; Khor et al. 2011) and, reduced gut mobility (loperamide) (Shi et al. 2014), suggesting shared, evolutionarily conserved biological functions related to pain and mediated by these receptors. While morphine primarily targets zMOP, it also shows affinity for zebrafish δ-opioid (zDOP) receptor (Rodriguez et al. 2000) expressed as two functional copies (zDOPa and zDOPb) (Barrallo et al. 1998; Pinal-Seoane et al. 2006), both widely spread throughout the brain, including regions related to analgesia and mood (Pinal-Seoane et al. 2006). Hence, the conservation of opioidergic circuitry between zebrafish and mammals reinforces the translational value of this model for investigating CNS-targeted analgesic mechanisms.

In contrast, nonsteroidal anti-inflammatory drugs (NSAIDs), such as diclofenac, inhibit both cyclooxygenase-1 (COX-1) and cyclooxygenase-2 (COX-2), which reduces prostaglandin synthesis associated with peripheral inflammation in humans (Leathers and Rogers 2023). Although zebrafish possess one copy of COX-1 (*ptgs1*) and two functional copies of COX-2 (*ptgs2a/ptgs2b*), only the latter two are related to the inflammatory and analgesic process (Leiba et al. 2023). Thus, both *ptgs2a* and *ptgs2b* contribute to peripheral pain signaling and represent key targets for NSAID-mediated analgesia, particularly in hyperalgesic and inflammatory conditions (Leiba et al. 2023). NSAIDs are effective in reducing pain by suppressing the production of pro-inflammatory prostaglandins, which are key mediators in triggering peripheral pain signaling (Leathers and Rogers 2023). Since the analgesic effects of diclofenac are peripheral (Leathers and Rogers 2023), it is expectable that it was less effective in correcting UCS-induced hyperalgesia, likely involving central modulation. Therefore, the central action of morphine highlights its dual role in managing both pain and mood, unlike diclofenac's peripheral effects.

Furthermore, the whole-body cortisol data support a pain-related phenotype after UCS exposure with AA injection, which is at least partly driven by activation of the stress axis. Animals exposed to UCS showed elevated cortisol levels (with and without AA injection), which were partly reduced by morphine injection. Likewise, AA 1.0% alone did not increase whole-body cortisol in unstressed fish, and its levels did not differ between the UCS-alone and UCS + AA 1.0% groups, indicating that stress exposure was the primary driver of endocrine activation in this paradigm. This result is consistent with activation of the hypothalamic–pituitary–interrenal (HPI) axis (homologous to the mammalian HPA axis) exposed to UCS, which raises circulating cortisol and lowers the pain threshold (Piato et al. 2011). The fact that morphine reduced both pain behavior and cortisol levels suggests an opioidergic modulation of stress responsiveness that contributes to the observed sensitization. For instance, HPI activation can lead to β-endorphin (β-END) release, which negatively modulates the HPI axis and decreases cortisol levels (Gonzalez-Nunez et al. 2003). Like morphine, β-END is also a µ-opioid receptor agonist, which can promote analgesic effects (Zaig et al. 2021). Also, our data are in line with previous findings showing an inhibitory effect of endogenous opioids on the HPA axis via both µ- and κ-opioid receptors in humans, further corroborating the influence of the opioidergic system on stress-related pathways (Kreek et al. 2005). Here, morphine reduced both writhing-like behavior and cortisol levels, supporting an opioidergic contribution to stress-related sensitization. However, causal directionality cannot be determined in the present design, meriting further studies. In summary, the behavioral and endocrine findings converge: UCS increases stress hormones and pain sensitivity, and the opioidergic intervention attenuates both. Together, these convergent effects strengthen the neuropharmacological interpretation of UCS-induced hyperalgesia as a centrally mediated stress–pain interaction.

Although this study provides behavioral evidence that UCS lowers the response threshold to AA injection in zebrafish, additional neurochemical or receptor-expression analyses can clarify mechanistic interpretation and help dissociate central from peripheral contributions. While the well-established doses of morphine (2.5 mg/kg) and diclofenac (40 mg/kg) follow standard zebrafish pain protocols and ensure comparability with previous data, a dose–response curve may offer additional pharmacological resolution in future studies. Likewise, sexes were pooled in the present study based on prior reports describing no sex differences in nociceptive or cortisol responses, a choice that enhances statistical power. However, targeted sex-specific analyses and further dissociation of locomotor from nociceptive outcomes can be valuable for extending these findings. Lastly, while molecular validation of receptor-specific mechanisms would provide additional resolution, the present study was designed to establish a functional neuropharmacological phenotype suitable for behavioral pharmacology and translational screening.

Importantly, the zebrafish model offers unique advantages for scalable drug discovery and high-throughput screening. Its small size, low maintenance costs, and compatibility with automated behavioral tracking enable rapid testing of multiple compounds and doses under controlled conditions (Kalueff et al. 2014; Stewart et al. 2014). The robust and quantifiable UCS-induced hyperalgesia phenotype described here, combined with validated pharmacological responsiveness to centrally acting agents and the whole-body cortisol measures, provides a reliable platform for identifying and prioritizing CNS-targeted analgesics. Such scalability underscores the potential of this model to bridge preclinical findings with early-phase drug development more efficiently than traditional mammalian systems. This scalability positions the model as a valuable intermediary between basic neuropharmacological research and preclinical analgesic development.

In summary, our findings demonstrate that UCS lowers the pain threshold in adult zebrafish, supporting the interplay between UCS-induced behavioral deficits and pain and also highlighting the relevance of zebrafish models for studying the relationship between pain and depression. Their modulation by morphine, but not diclofenac, suggests central rather than peripheral mechanisms of this phenotype. Beyond their neuropharmacological relevance, these findings establish a robust and quantifiable stress-induced hyperalgesia phenotype suitable for preclinical drug discovery, particularly for screening CNS-targeted analgesics, and provide a tractable platform for probing the neurobiological basis of stress–pain comorbidity. Finally, our results support the use of zebrafish as a translational model for investigating depression- and pain-responses in vivo, as well as to screen novel putative CNS drugs that target both domains individually and/or jointly.

## Supplementary Information

Below is the link to the electronic supplementary material.

Supplementary Material 1

## Data Availability

No datasets were generated or analysed during the current study.

## Abbreviations

AA: Acetic acid
AUC: Area under the curve
β-END: β-endorphin
CEUA: Ethics Committee on the Use of Animals
CNS: Central nervous system
CONCEA: National Council for the Control of Animal Experimentation
COX-1: Cyclooxygenase-1
COX-2: Cyclooxygenase-2
CTRL: Control
DOP: Delta-opioid receptor
DS: Diclofenac sodium
HPA: Hypothalamic-pituitary-adrenal axis
HPI: Hypothalamic-pituitary-interrenal axis
i.p.: Intraperitoneal
MOP: Mu-opioid receptor
NIH: National institutes of health
NSAIDs: Nonsteroidal anti-inflammatory drugs
PBS: Phosphate-buffered saline
UCS: Unpredictable Chronic Stress
zDOPa: Zebrafish δ-opioid receptor type a
zDOPb: Zebrafish δ-opioid receptor type b
zMOP: Zebrafish µ-opioid receptor
ZBC: Zebrafish behavioral catalogue
