# Supplementary material for: Opioidergic modulation of stress-induced hyperalgesia in adult zebrafish
Source: J Neural Transm (Vienna). 2026 May 26;133(7):1739–54. doi: 10.1007/s00702-026-03159-3 (PMC13428717; doi:10.1007/s00702-026-03159-3)
Supplement: Supplementary file 1 — Supplementary Material 1 [file 702_2026_3159_MOESM1_ESM.docx]

## **Supplementary Information**

Opioidergic modulation of stress-induced hyperalgesia in adult zebrafish

Fabiano V. Costa^a,b,c, h*^, Lana Ferreira^b^, Lucca K. Lima^b^, Julia Canzian^d,e^, Allan V. Kalueff ^f,g^,

Denis B. Rosemberg^d,e,g^, Carla D. Bonan^a,b,c^

^a^Graduate Program in Cellular and Molecular Biology, School of Health and Life Sciences, Pontifical Catholic University of Rio Grande Do Sul, Porto Alegre, RS, Brazil.

^b^Laboratory of Neurochemistry and Psychopharmacology, School of Health and Life Sciences, Pontifical Catholic University of Rio Grande do Sul, Porto Alegre, RS, Brazil.

^c^Graduate Program in Medicine and Health Sciences, School of Medicine, Pontifical Catholic University of Rio Grande Do Sul, Porto Alegre, RS, Brazil.

^d^Laboratory of Experimental Neuropsychobiology, Department of Biochemistry and Molecular Biology, Natural and Exact Sciences Center, Federal University of Santa Maria. Santa Maria, RS, Brazil.

^e^Graduate Program in Biological Sciences: Toxicological Biochemistry, Federal University of Santa Maria, Santa Maria, RS, Brazil.

^f^Department of Biosciences and Bioinformatics, Suzhou Municipal Key Laboratory of Neurobiology and Cell Signaling, Xi’an Jiaotong-Liverpool University, Suzhou, China

^g^International Zebrafish Neuroscience Research Consortium (ZNRC), New Orleans, USA

^h^Undergraduate Program in Nursing, La Salle University, Canoas, RS, Brazil

^*^Correspondence to: Fabiano V. Costa, Ph.D., Pontifical Catholic University of Rio Grande do Sul, Ipiranga Avenue, 6681, Building 12D/301.Porto Alegre, RS, Brazil. [fvcosta88@gmail.com](mailto:fvcosta88@gmail.com)


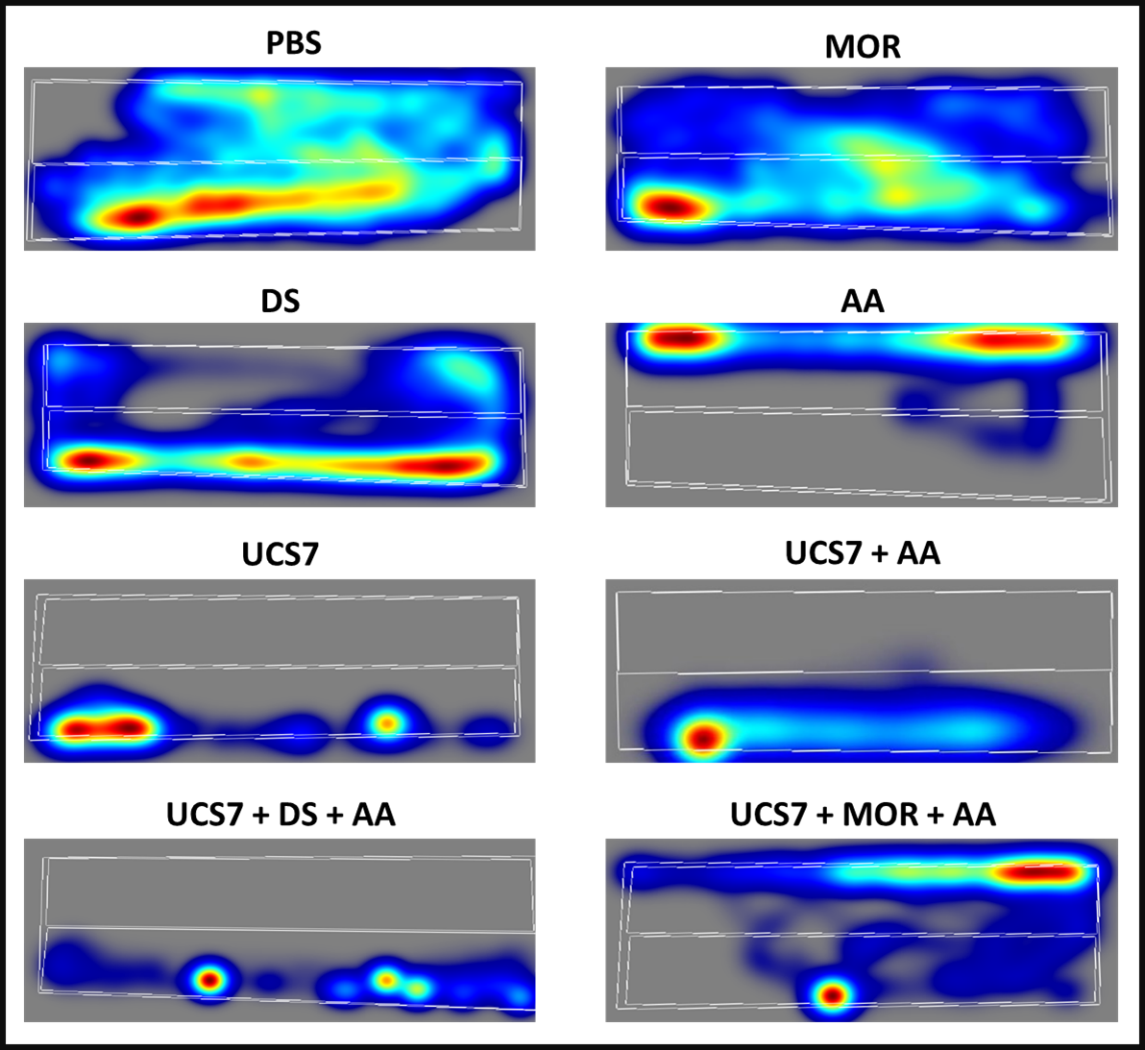

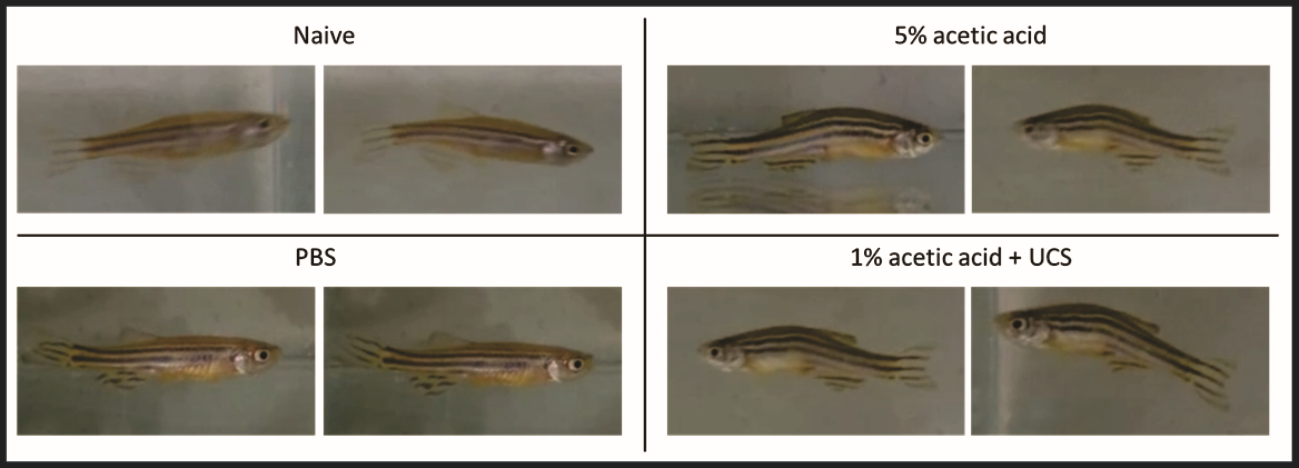
**Supplementary Fig. 1.** Representative images displaying the zebrafish phenotypes after acetic acid injection, as well as the effects of acetic acid plus UCS protocol in relation to PBS group.

**Supplementary Fig. 2.** Representative heatmap of zebrafish locomotor profiles under main treatments.
